# Northern Ohio Dental Association

**Published:** 1870-06

**Authors:** H. H. Newton


### Proceedings of Societies.

### NORTHERN OHIO DENTAL ASSOCIATION.

CLEVELAND, OHIO, May 3d, 1870—10 o'clock, A. M.

The Association met according to the requirements of the Constitution.

Meeting called to order by the President, Dr. Whitslar.

Minutes of the last meeting read and approved.

Members present—Drs. B. Strickland, C. R. Butler, F. S. Slosson, B. T. Spelman, C. Palmer, W. P. Horton, H. H. Newton, J. F. Siddall, L. Buffett, Chas. Buffett, F. S. Whitslar, H. L. Ambler, E. J. Way, B. F. Robinson, J. E. Robinson, S. B. Burnham, C. B. Knowlton, J. C. Whinnery.

Moved and carried that the report of the Executive Committee be adopted.

Two o'clock, P. M.—Discussions being in order, the President called for the first article on discussions, viz: "Dental Quackery and its Remedies."

Dr. Spelman remarked that he was sorry to see the article named as one of the subjects for discussions; thought the true motive for each operator was to regulate his practice according to the rules of justice and his own conscience, and leave the result to a higher power.

Dr. L. Buffett thought the subject one of sufficient importance in its bearings upon the profession; that some effort should be made to purge out of our noble calling such quackery that brings only disgrace upon the profession.

Dr. Strickland remarked that there was now no excuse for any person entering our profession without such preparation as would enable him to practice in a scientific manner, and therefore no excuse for an ignorant quack. As to the learned quack, he would exclude him from the practice of his profession by the aid of the law.

Dr. Siddall remarked that he thought it to be the duty of all good Dentists to assist the uninformed and thereby elevate them.

Dr. Siddall read an essay on "Cast Metallic Base."

Dr. Strickland moved that a vote of thanks be tendered to Dr. Siddall for the able and instructive paper, and that it be received and placed on file.

The association then passed to the discussion of "Cast Metallic Base."

Dr. Newton stated that he had some experience with Weston's metal, and was not satisfied with the result; found that the plates would break in the mouth by mastication; considers it very brittle, and therefore unfit for Dental purposes.

Dr. Strickland presented a plate made of an alloy of silver and platina, with a solder for uniting the teeth to the plate by casting, made by Drs. Watt and Williams.

Dr. Spelman advocated the use of some of the alloys of tin as a substitute for rubber.

Dr. Horton was glad that some effort was being made by the members of the profession to supplant rubber, and thanked those who were engaged in so laudable an undertaking.

Dr. Slosson read an essay on "Atrophy." It was moved that the thanks of the association be extended to Dr. Slosson and his essay be placed on file.

### SECOND DAY.

### *Morning Session.*

The resolution offered by Dr. Strickland, one year ago, was amended thus:

*Resolved*, It shall be the duty of the Secretary to strike out from the list of active members the name of any person who has failed to participate in the meetings of the Association, or pay his dues to the same, for two successive years, after being duly notified thereof that such dues have been assessed, and the person losing his membership shall not be restored again except by a vote of two-thirds of all members present at any regular meeting. Carried.

The hour for the election of officers for the incoming year had now arrived. The Chair appointed Drs. Siddall and Newton as tellers, and then proceeded to the election, which resulted as follows:

President—Dr. J. E. Robinson.

Vice President—Dr. E. J. Way.

Treasurer—Dr. C. R. Butler.

Recording Secretary—Dr. H. H. Newton.

Corresponding Secretary—Dr. W. P. Horton.

Examining Committee—Drs. L. Buffett, C. R. Butler, C. Palmer.

Delegates to the American Association—Drs. J. C. Whinnery, F. S. Whitslar, C. R. Butler, C. Palmer, E. J. Way.

Alternates—Drs. Chas. Buffett, W. P. Horton, H. H. Newton, J. F. Siddall, F. S. Slosson.

Committee on Dental Ethics—Drs. Slosson, Way, and Ambler.

The hour had now arrived for the newly-elected President, Dr. J. E. Robinson, to take the Chair. Drs. F. S. Slosson and B. F. Robinson were appointed to conduct him to the Chair, and in a few appropriate remarks he thanked the association for the honor conferred and the confidence they reposed in him, and would endeavor to discharge the duties to the best of his ability.

Dr. Whitslar, the retiring President, read a very able and interesting address, which was listened to with marked attention.

Dr. Horton moved that the address be obtained for publication. Carried.

Moved to adjourn at 2 o'clock P. M.

Met at the hour appointed. Meeting called to order by the President.

Subject for discussion: "Diseases of the Gums and Alveolus and their Treatment."

Dr. Horton had used the actual cautery in several cases with marked success, especially of fungeous grath.

Dr. L. Buffett remarked that he should much prefer other means in such cases; had no doubt it would be successful, but thought there might be danger of destroying the nerves of the teeth from congestion.

Dr. Strickland said if he were to use the cautery at all, it would be in connection with a galvanic battery.

Dr. Slosson said he was called in consultation with Dr. Ackly to a young lady afflicted with hypertrophy of the upper and lower jaws; extracted part of the teeth of both jaws, and cut away part of the gums and process, and used the cautery, but did not know the result. In another case, used nitrate of silver with good results.

*Miscellaneous.*—Dr. C. R. Butler called the attention of the association to the use of rubber dam, first used by Dr. S. D. Barnum. Said he thought the profession was under many obligations to him for its successful introduction. Said he could operate in cases where he could not control the saliva with the use of napkins; considers it a great acquisition to the Dentist, and would not like to be deprived of its use.

Dr. Strickland confirmed the ideas of Dr. Butler in its value to the profession, and thought that every Dentist would be amply paid for the time it takes to put it on.

Dr. Ambler uses the rubber dam with success, and would not like to be without it; uses scissors to cut the holes instead of a punch; says they are quite as good.

Moved we take up the subject of heavy foils and heavy mallets.

Dr. C. Palmer has experimented with No. 16 heavy foil with success; uses a five or six ounce mallet; finds no inconvenience to his patients in its use.

Dr Butler likes heavy foil for surface work; gives him more satisfaction than lighter foils; but remarked that we must be absolutely protected against moisture; uses shallow serrations upon his instruments, and a combination mallet of wood and metal—weight four to five ounces; does not think that heavy mallets produce any more soreness than light ones; has seen operations made with 120 foil with success; has used No. 20 to 60 folds in mats one-eight inch wide, four to eight folds in thickness; it is easily managed, and can finish fillings much quicker with it.

Dr. Horton has only used No. 6 foil, and until recently a light mallet; has found the heavy ones gives more satisfaction to his patients and less pain.

Dr. Ambler uses a heavy mallet and a light one, but prefers the heavy; has used foil made in Pittsburg, Pennsylvania, of a fine quality of Nos. 12 and 20, but not as tough as Johnston's.

On motion it was decided to meet at Put-in-Bay Island, on the 1st Tuesday in May, 1871.

Meeting adjourned.

H. H. Newton, Recording Secretary.

### THE AMERICAN DENTAL ASSOCIATION.

Dr. Taft—*Dear Sir:* The Committee of Arrangements have made the following arrangements for the next meeting to be held in Nashville in August next. Dr. Morgan has secured the use of the Representative Hall for holding the meetings, and has secured a reduction of fare at the Maxwell House, one of the finest hotels on the continent, from the regular rates to from $2 50 to $3 50 per day—owing to the floor occupied. The Committee of Five appointed at the last annual meeting to secure half fare on the various railroads, have as yet made no report.

